# Ubiquitin Proteasome Gene Signatures in Ependymoma Molecular Subtypes

**DOI:** 10.3390/ijms232012330

**Published:** 2022-10-15

**Authors:** Jerry Vriend, Thatchawan Thanasupawat, Namita Sinha, Thomas Klonisch

**Affiliations:** 1 Department of Human Anatomy and Cell Science, Rady Faculty of Health Sciences, University of Manitoba, Winnipeg, MB R3E 0J9, Canada; 2 Department of Pathology, Rady Faculty of Health Sciences, University of Manitoba, Winnipeg, MB R3E 3P5, Canada; 3 Department of Medical Microbiology and Infectious Diseases, Rady Faculty of Health Sciences, University of Manitoba, Winnipeg, MB R3E 0J9, Canada; 4 Children’s Hospital Research Institute of Manitoba, Winnipeg, MB R3E 3P4, Canada; 5 CancerCare Manitoba, Winnipeg, MB R3E 0J9, Canada

**Keywords:** ependymoma, ubiquitin proteasome system, neddylation, sumoylation, APC/c complex, conjugases, ligases

## Abstract

The ubiquitin proteasome system (UPS) is critically important for cellular homeostasis and affects virtually all key functions in normal and neoplastic cells. Currently, a comprehensive review of the role of the UPS in ependymoma (EPN) brain tumors is lacking but may provide valuable new information on cellular networks specific to different EPN subtypes and reveal future therapeutic targets. We have reviewed publicly available EPN gene transcription datasets encoding components of the UPS pathway. Reactome analysis of these data revealed genes and pathways that were able to distinguish different EPN subtypes with high significance. We identified differential transcription of several genes encoding ubiquitin E2 conjugases associated with EPN subtypes. The expression of the E2 conjugase genes *UBE2C*, *UBE2S*, and *UBE2I* was elevated in the ST_EPN_RELA subtype. The UBE2C and UBE2S enzymes are associated with the ubiquitin ligase anaphase promoting complex (APC/c), which regulates the degradation of substrates associated with cell cycle progression, whereas UBE2I is a Sumo-conjugating enzyme. Additionally, elevated in ST_EPN_RELA were genes for the E3 ligase and histone deacetylase *HDAC4* and the F-box cullin ring ligase adaptor *FBX031*. Cluster analysis demonstrated several genes encoding E3 ligases and their substrate adaptors as EPN subtype specific genetic markers. The most significant Reactome *Pathways* associated with differentially expressed genes for E3 ligases and their adaptors included antigen presentation, neddylation, sumoylation, and the APC/c complex. Our analysis provides several UPS associated factors that may be attractive markers and future therapeutic targets for the subtype-specific treatment of EPN patients.

## 1. Introduction

Ependymomas (EPN) are glial neoplasms thought to arise from primitive ependymal lining cells of the ventricles in the brain and spinal cord of the central nervous system (CNS) and mainly occur in younger children and younger to mid-age adults. EPN account for 2–3% of primary brain tumors but 8–10% of all pediatric brain tumors. Many EPN patients present with hydrocephalus as this tumor may block the flow of cerebrospinal fluid. Based on their location within the CNS, EPN are divided into supra-tentorial (ST), posterior fossa (PF), and spinal cord (SP) groups. Each of these three EPN groups presents with three different molecular subtypes and some of which are associated with distinct oncogenic mechanisms and drivers. Adding to this complexity, all nine different EPN molecular subtypes have different age and gender distributions and prognostic values [1]. The EPN subtypes may originate from different progenitor cells that includes radial glial cells [2].

The ubiquitin proteasome system (UPS) serves to regulate proteasomal degradation of proteins and can also contribute to transcriptional regulation of genes [3]. The UPS contributes to the regulation of all major cellular processes, including signal transduction, cell cycle, growth, development, differentiation, metabolism, synaptic regulation, antigen presentation, apoptosis, and autophagy. In the present study, we have employed publicly available datasets to investigate the expression profiles of genes encoding components of the UPS in different molecular subtypes of human supratentorial, posterior fossa, and spinal EPN subgroups. Differential gene expression between the EPN molecular subtypes was determined by analysis of variance (ANOVA) through the R2 Genomics site.

## 2. Methods

We have analyzed a publicly available gene expression dataset on EPN molecular subtypes through the R2 Genomic analysis and visualization platform (https://r2.amc.nl). The expression of genes encoding UPS associated factors was compared between 8 subtypes of 209 EPN samples in the Pfister dataset (GEO ID: GSE64415). These EPN subtypes were classified as posterior fossa EPN a (PF_EPN_A), posterior fossa EPN b (PF_EPN_B), posterior fossa subependymoma (PF_SE), spinal cord EPN (SP_EPN), spinal cord myxopapillary ependymoma (SP-MPE), supratentorial EPN Rela (ST_EPN_RELA), supratentorial EPN Yap1 (ST_EPN_YAP1), and supratentorial subependymoma (ST_SE) [4]. The Pfister dataset did not include microarray data on the spinal sub-ependymoma (SP-SE) subgroup due to lack of availability of RNA from these samples. Analysis of variance of log2 expression data was used to determine significant differential gene expression. Unsupervised cluster analysis and illustration of clusters of UPS genes encoding E3 ligases and E3 ligase adaptors in the various EPN subtypes was done with the Morpheus heat map program (Morpheus software, https://software.broadinstitute.org/morpheus). The Cytoscape (version 3.8) with ClueGO V2.5.9 plug-in was used to identify pathways over-represented in the list of genes encoding E3 ligases and E3 ligase adaptors, genes associated with these pathways, and network analysis. Our requirements for the definition of a Gene Ontology (GO) group were a minimum of 3 genes and at least 4% of the genes assigned to a biological pathway. The French glioma dataset, accessible through the R2 genomics site, was used to generate survival curves associated with the expression of selected genes. In the Pfister dataset, poorest overall survival was reported for the EPN-A and ST-EPN-RELA subtypes [4]. Survival data are not available in the R2 genomics dataset for this dataset.

## 3. Results

The Pfister EPN dataset included age at diagnosis in 88.5% (185 of 209) of cases (Table 1). For the PF-EPN-A and ST-EPN-RELA subtype, 83% and 75% of cases were between the ages of 0–10 years of age, respectively, whereas for the PF-EPN-B group all cases were >10 years of age. Because of the small sample size of the remaining groups, generalized statements about age of disease manifestation could not be made with confidence. The age distribution in the subtypes of EPN in this dataset is shown in Table 1.

### 3.1. E1 Ubiquitin Activator Gene Expression in EPN Subtypes

**Figure 1:**
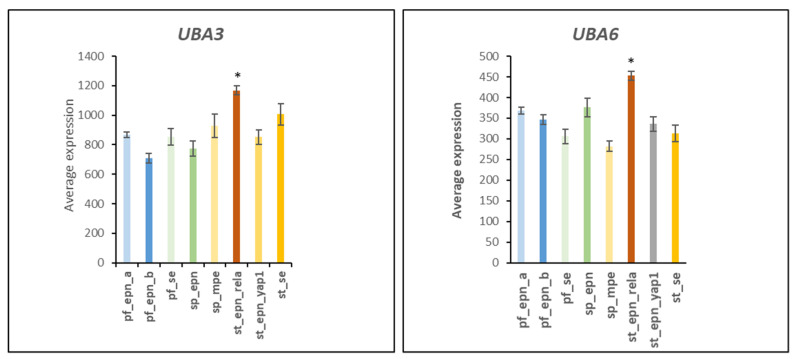
Differential expression of genes encoding E1 ubiquitin activating enzymes by Anova, *UBA3* (F = 20.60, *p* = 9.29 × 10^−21^) and *UBA6* (F = 14.19, *p* = 6.22 × 10^−15^) in EPN subtypes. * Expression of *UBA3* and *UBA6* were significantly elevated in ST_EPN_RELA compared to all other EPN subtypes by at least *p* < 0.01 (with the exception of SP_MPE for *UBA3*) as determined by *t*-test. Dataset GSE64415.

The E1 ubiquitin activator enzymes of the UBA1 family transfer ubiquitin to multiple E2 conjugases [5]. Gene expression of *UBA3* (F = 20.60, *p* = 9.29 × 10^−21^) and *UBA6* (F = 14.19, *p* = 6.22 × 10^−15^) was highly significantly elevated in ST_EPN_RELA (Figure 1) as compared to the other EPN subtypes. By contrast, differential expression of the UBA1 gene in EPN subtypes was statistically not very significant (F = 3.31, *p* = 2.37 × 10^−3^). The UBA3 (NEDD8 Activating Enzyme E1 subunit 2, aka NAE2) protein is part of the neddylation pathway [6] and forms a dimer with NAE1 [7], which transfers Nedd8 to the ubiquitin E2 conjugases UBE2M and UBE2F [6]. The ubiquitin conjugases, in turn, transfer Nedd8 to a variety of ubiquitin E3 ligases, including Cullin ring ligases which are activated by NEDD8 [8]. The UBA6 protein specifically transfers ubiquitin to the E2 ubiquitin conjugase USE1 (aka UBE2Z, aka UBA6-specific E2 conjugating enzyme) [9].

### 3.2. Ubiquitin Conjugase (E2) Gene Expression in EPN Subtypes

In the Pfister data set, 31 genes encoding ubiquitin E2 conjugases were differentially expressed (*p* < 0.0001, log 2 data). Expression of several E2 conjugase encoding genes was elevated in the ST_EPN_RELA subgroup compared to the other seven groups, including *UBE2C*, *UBE2S*, *UBE2T*, *UBZE2Q1*, *UBE2V2*, *UBE2D2 and UBZE2J2* (*p* < 0001 by Anova). Expression of *UBE2E3* was specifically elevated in PF-EPN_A, while expression of *UBE2Z* was specifically elevated in SP-MPE (*p* < 0.0001). Reactome analysis of the 31 differentially expressed ubiquitin E2 conjugase genes identified the immune system as the main Reactome *Pathway* associations with these genes (Table 2). The Reactome *Pathway* terms represented by over 80% (25/31) of these genes included *“Antigen processing: ubiquitination and proteasome degradation”* and *“Class I MHC mediated antigen processing and presentation”* (Group *p* value for over-expression = 4.95 × 10^−35^). We concluded that the expression of these ubiquitin E2 conjugase genes was differentially stimulated by one or more transcription factors associated with the immune response.

**Figure 2:**
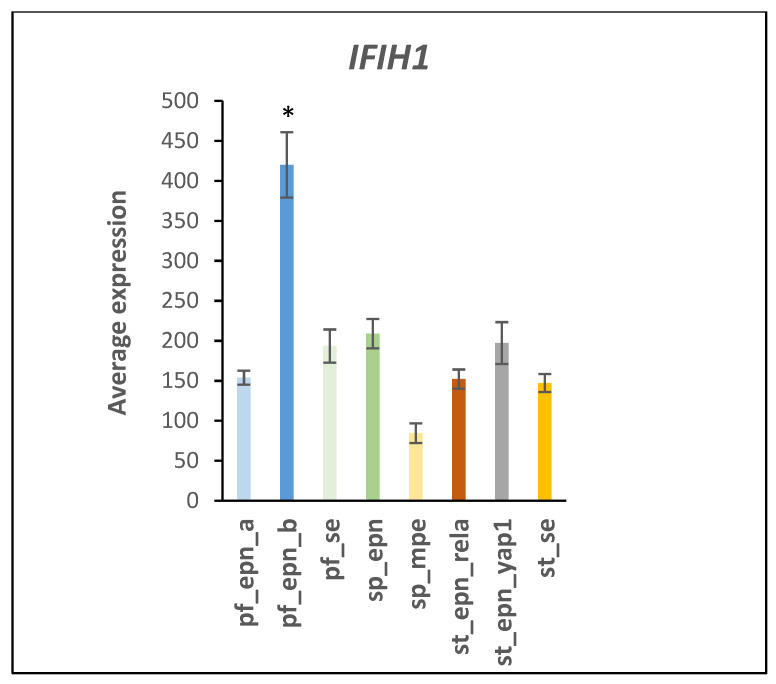
Increased *IFIH1* expression in the PF_EPN_B subtype (by Anova, F = 19.36, *p* = 1.08 × 10^−19^). * significantly different from all other groups by *t*-test *p* < 0.01. Dataset GSE64415.

Reactome pathway analysis of the EPN data revealed differential expression of four genes encoding ubiquitin E2 conjugases (*UBE2D1*, *UBE2D2*, *UBE2K*, *UBE2L6*) that were associated with the DDX58/IFIH1 signaling pathway (Table 2). The expression of the *DHX58* gene was specifically elevated in the ST_EPN_RELA subtype (F = 10.82, *p* = 1.48 × 10^−11^). *DHX58* encodes a regulator for DDX58 (aka DExH-Box Helicase 58 or RIG1) which is an innate immune receptor that initiates proinflammatory and type I interferon induced signaling pathways in response to viral infections [10,11]. *IFIH1* (Interferon Induced with Helicase C domain 1) expression was specifically elevated in the PF_EPN_B subtype (Figure 2) and is part of an interferon response signature [12].

**Figure 3:**
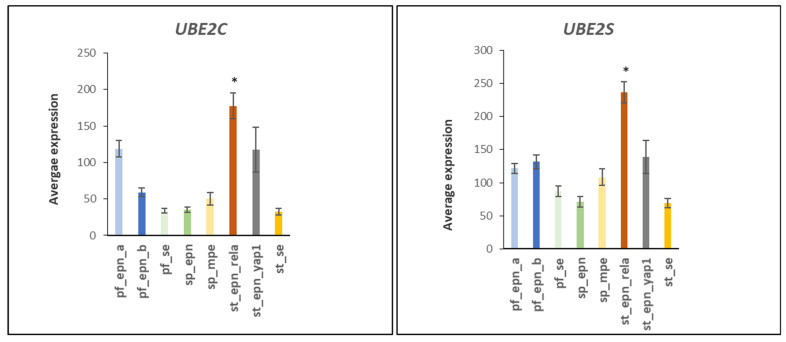
Expression of *UBE2C* and *UBE2S* is significantly increased in the ST_EPN_RELA subtype (F = 9.92, *p* = 1.28 × 10^−10^; F = 16.00, *p* = 1.19 × 10^−16^). * *UBE2C*-all significantly higher compared to other groups by *t*-test except *ST*-*EPN_YAP1 group* * *UBE2S*-all significantly higher compared to each of the other groups by *t*-test. Dataset GSE64415.

**Figure 4:**
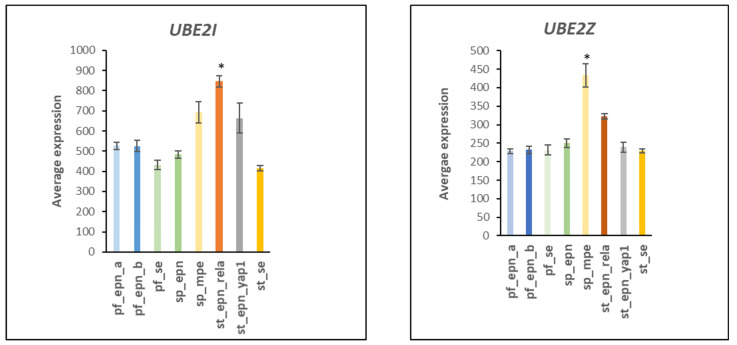
* By *t*-test *UBE2I* mean of the St_Epn_Rela group is significantly greater (by Anova, F = 22.36, *p* = 3.06 × 10^−22^) than that of all other groups by at least *p* < 0.05. The most significant difference was between the PF-EPN-A and the RELA groups (t = 9.88, *p* < 0.0001). By *t*-test mean of *UBE2Z* SP_MPE is significantly greater than that of all other groups, *p* < 0.0001. By Anova of *UBE2Z*, F = 28.73, *p* = 3.14 × 10^−27^. Dataset GSE64415.

Four differentially expressed ubiquitin E2 conjugase genes (*UBE2C*, *UBE2S*, *UBE2D1*, *UBE2E1*) contributed to 22 Reactome *pathways* statistically associated with the anaphase promoting complex/cyclosome (APC/c) pathway (Table 2) and to 37 Reactome *Reactions* (Table 3). *UBE2C* (aka *UBCH10*) and *UBE2S* work in tandem [13,14,15] and their role in cell cycle progression has been well documented [13,14,15]. UBE2C initiates ubiquitination of the APC/c E3 ligase complex, while UBE2S elongates ubiquitin chains on the APC/c complex initiated by UBE2C and enhances the ability of UBE2C to initiate ubiquitination [14,15,16]. Increased expression of *UBE2C*, and *UBE2S* in the ST_EPN_RELA subtype is shown in Figure 3. Relatively higher expression of *UBE2I*, a SUMO conjugating enzyme, in the ST_EPN_RELA subgroup, and higher expression of *UBE2Z* in the SP_MPE subgroup is shown in Figure 4.

### 3.3. Ubiquitin E2 Conjugases and Patient Survival Times

In addition to *UBE2C* and *UBE2S*, the ST_EPN_RELA subtype demonstrated the over-expression of several other ubiquitin E2 conjugases, including *UBE2I* (*p* = 3.06 × 10^−22^ by Anova), *UBE2J2* (*p =* 5.32 × 10^−19^), *UBE4B* (*p =* 4.23 × 10^−19^), *UBE2T* (*p =* 6.50 × 10^−8^), *UBE2Q1* (*p =* 8.06 × 10^−10^), *UBE2D2* (*p =* 5.10 × 10^−12^), and *UBE2V2* (*p =* 2.03 × 10^−8^). We used the French glioma dataset to examine the association of over-expressed ubiquitin E2 conjugases with patient survival data. Shorter survival was associated with high expression levels of *UBE2C* (chi = 29.87, *p* = 4.6 × 10^−8^), *UBE2S* (chi = 24.27, *p* = 8.4 × 10^−7^), *UBE2J2* (chi = 39.89, *p* = 2.7 × 10^−10^) and *UBE2T* (chi = 21.96, *p* = 2.8 × 10^−6^). High expression of *UBE2Z* was associated with shorter survival in SP_MPE (chi = 16.05, *p* = 6.2 × 10^−5^).

### 3.4. Ubiquitin E3 Ligase Gene Expression in EPN Subtypes

**Figure 5:**
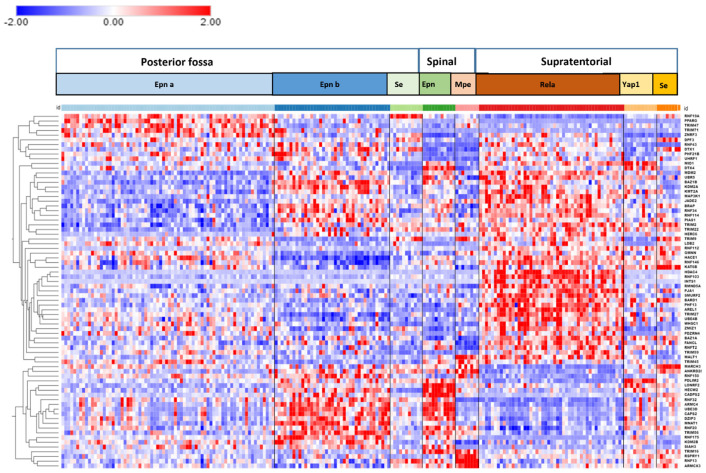
Cluster analysis of differentially expressed ubiquitin E3 ligase genes in different EPN subtypes. Genes are listed again in the order they appear in the heatmap (top to bottom) for better readability. Genes on the right of the heatmap are listed again for better visibility: *RNF19A*, *PPARG*, *TRIM47*, *TRIM71*, *ZNRF3*, *DPF3*, *RNF43*, *DTX1*, *PHF21B*, *UHRF1*, *MID1*, *DTX4*, *MDM2*, *UBR5*, *BAZ1B*, *KDM2A*, *KMT2A*, *MAP3K1*, *JADE2*, *BRAP*, *RNF34*, *RNF114*, *PIAS1*, *TRIM2*, *TRIM22*, *HERC6*, *TRIM9*, *LDB2*, *RNF112*, *GMNN*, *HACE1*, *RNF146*, *KAT6B*, *HDAC4*, *RNF103*, *INTS1*, *RMND5A*, *PJA1*, *SMURF2*, *BARD1*, *PFH13*, *AREL1*, *TRIM27*, *UBE4B*, *WHSC1*, *ZMIZ1*, *PDZRN4*, *BAZ1A*, *FANCL*, *RNFT2*, *TRIM59*, *MALT1*, *TRIM45*, *MARCH3*, *ANKRD28*, *RNF150*, *PDLIM2*, *LONRF2*, *HECW2*, *CADPS2*, *RNF32*, *ARMC4*, *UBE3D*, *CAPS2*, *DZIP3*, *MNAT1*, *RNF20*, *TRIM56*, *RNF175*, *KDM2B*, *SIAH3*, *TRIM16*, *RSPRY1*, *RNF13*, *ARMCX3*.

Differentially expressed genes encoding E3 ligases were identified through the R2 genomics platform in the Pfister dataset by Anova. Expression of the 75 most significantly different E3 ligase genes was used in the statistical analysis. The heatmap in Figure 5 shows the clusters of E3 ligase genes associated with different EPN subtypes. Table 4 and Table 5 summarize the statistically most significant Reactome *Pathways* and Reactome *Reactions* associated with the expression of the E3 ligase genes and identified the E3 ligase genes associated with these pathways. We identified several differentially expressed genes encoding ubiquitin E3 ligases by Reactome analysis as part of the immune response pathway in EPN.

**Figure 6:**
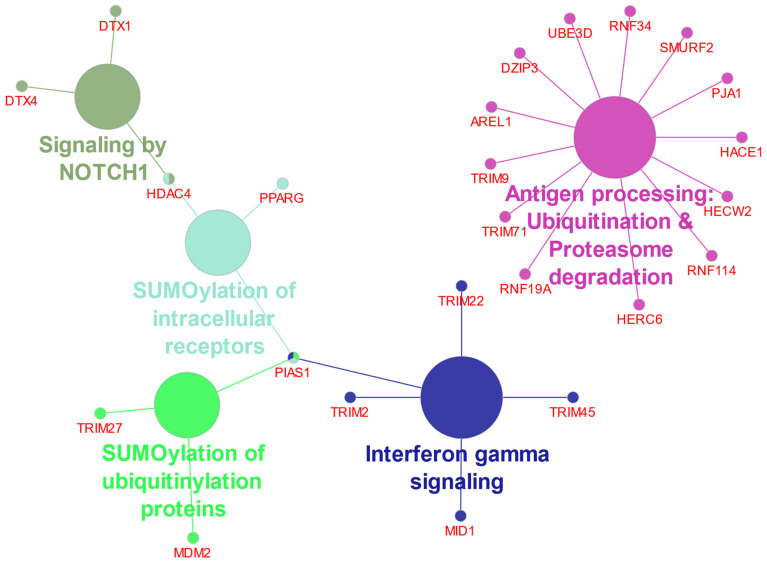
Reactome network of ubiquitin E3 ligase genes in ependymoma.

The most significant Reactome *Pathway* in the 75 most significant differentially expressed ubiquitin E3 ligases in the Pfister EPN dataset, as determined by the Reactome program ClueGo application, was *“Antigen processing: ubiquitination & proteasome degradation”*. The 13 genes identified in this pathway were the same as the 13 genes identified in the *“Class I MHC mediated antigen processing & presentation* pathway*”*. Because our cutoff point was 4% of the total genes in the pathway, only the first pathway was included in Table 4. The data suggested a role for several ubiquitin E3 ligases in Class I MHC mediated antigen processing. The Reactome network created by the GlueGo application is shown in Figure 6.

**Figure 7:**
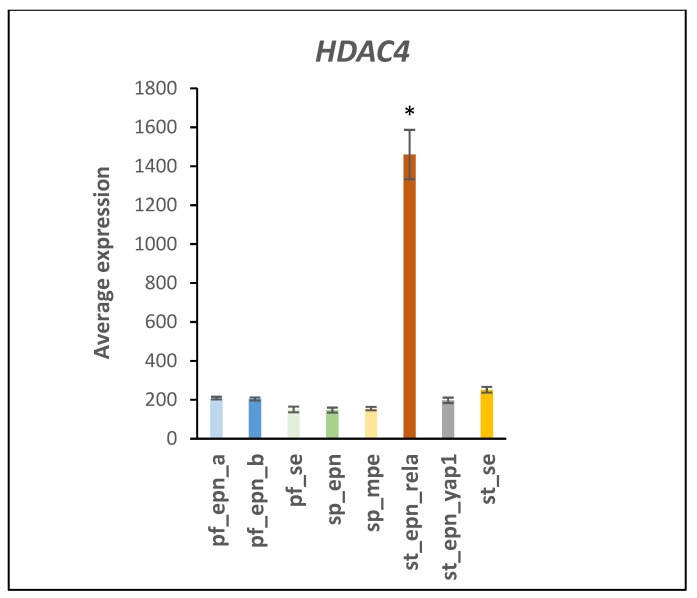
Significantly increased expression of the *HDAC4* gene in the ST_EPN_RELA subtype (by Anova, F = 44.88, *p* = 7.86 × 10^−38^). * by *t*-test significantly different from all other groups *p* < 0.001. Dataset GSE64415.

*HDAC4* (Histone deacetylase 4) was the most significantly up-regulated ubiquitin E3 ligase gene in the ST_EPN_RELA subtype (F = 44.88, *p* = 7.86 × 10^−38^) (Figure 7; see also heatmap in Figure 5). Multifunctional *HDAC4* encodes a protein that can act as a histone deacetylase and epigenetic regulator of transcription as confirmed by our GO analysis of the EPN dataset, which identified *HDAC4* as the most differentially expressed gene of all genes in the GO category of *“Epigenetic regulation of gene expression”*. However, the HDAC4 protein also contains a ubiquitin ligase domain which exhibits SUMO E3 ligase activity and is therefore included in the heatmap of E3 ligases (Figure 5) [17,18,19].

Illustrated in Figure 6, is the Reactome network “Analysis of the Gene Ontology (GO)” pathways emerging from the list of 75 top ubiquitin E3 ligase genes. It depicts the relationship between Notch signaling, HDAC4, and SUMOylation of intracellular receptors in the cluster of differentially expressed E3 ligase genes of Figure 5.

**Figure 8:**
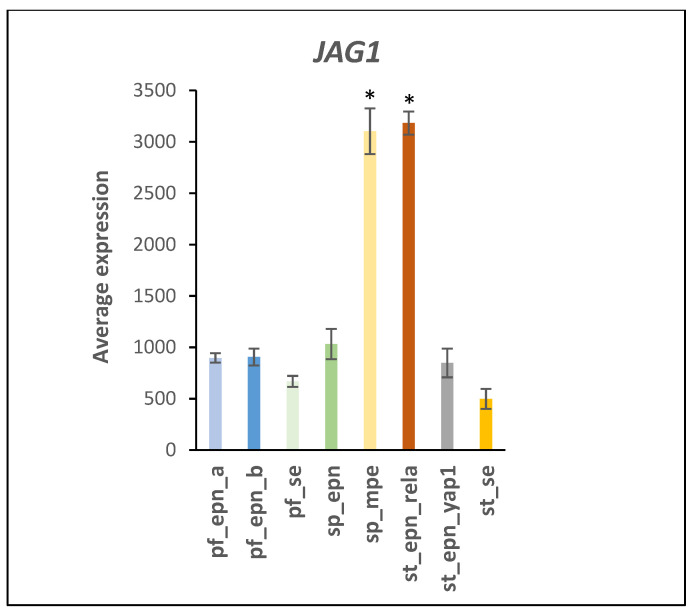
Over-expression of *JAG1* in SP_MPE and ST_EPN_RELA subtypes (by Anova, F = 108.15, *p* = 1.23 × 10^−64^). * significantly different from all other groups not indicated by *, at *p* < 0.001. Dataset GSE64415.

The expression of several Notch target genes [20,21] was elevated in specific EPN subtypes. Among these Notch target genes, expression of *CCND1* (F = 51.39, *p* = 1.77 × 10^−44^), *HES4* (F = 59.02, *p* = 2.11 × 10^−45^), *HES5* (F = 18.03, *p* = 1.65 × 10^−18^), *NRARP* (F = 28.96, *p* = 2.11 × 10^−27^), *ITPKC* (F = 78.02, *p* = 7.27 × 10^−54^), *CACNA1H* (F = 27.53, *p* = 2.48 × 10^−26^), and *SLC12A7* (F = 79.98, *p* = 1.19 × 10^−54^) was specifically elevated in the ST_EPN_RELA subtype. *HES5* and *CACNA1H* gene expression was increased approximately 5-fold and 15-fold in ST_EPN_RELA compared to the other subtypes, respectively. *CHST7* (F = 84.85, *p* = 1.50 × 10^−56^), *CTTNBP2* (F = 81.50, *p* = 2.96 × 10^−55^), *DKK1* (F = 59.16, *p* = 1.81 × 10^−45^, approximately 35-fold), *PDGFRA* (F = 41.76, *p* = 5.67 × 10^−36^), and *ALCAM* (F = 39.27, *p* = 1.97 × 10^−34^) were among those Notch target genes with elevated expression in SP-MPE. *JAG1* expression was equally over-expressed in both the ST_EPN_RELA and SP_MPE subtype (F = 108.15, *p* = 1.23 × 10^−64^) (Figure 8).

Our analysis of the Pfister EPN dataset on proteins with E3 ligase domains suggests a subtype-specific contribution of Notch signaling to the development of EPN. In support of these data, a recent study of de Almeida Magalhaes et al. [22] has documented the overexpression of *NOTCH1*, *JAG1*, *JAG2*, and *HES4* in the ST_EPN_RELA subgroup.

### 3.5. Differentiation and DNA Repair Factors Are UPS Targets in Selected EPN Subtypes

**Figure 9:**
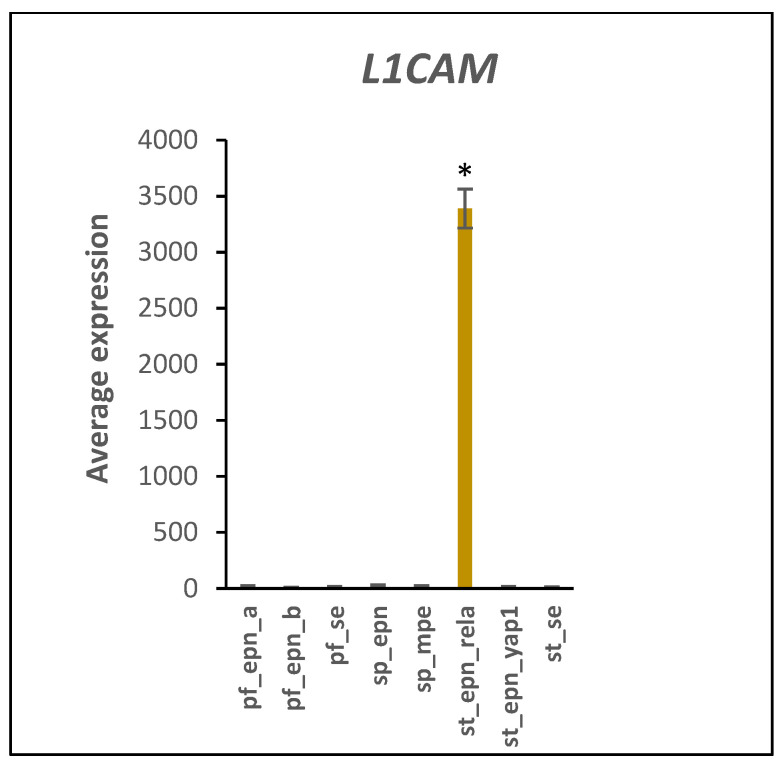
Gene encoding stem cell marker and cell adhesion factor, *L1CAM*, overexpressed in ST_EPN_RELA (by Anova, F = 173.55, *p* = 1.35 × 10^−81^). * significantly different from all other groups at *p* < 0.001. Dataset GSE64415.

The Pfister EPN dataset showed a selective increase in expression of the tumor invasion-promoting *L1CAM* gene [23] in the ST_EPN_RELA subtype at a very high level of significance (by Anova, F = 175, *p* = 1.35 × 10^−81^) (Figure 9). The *L1CAM* encodes a protein which may serve as a marker for the SP_EPN_RELA subtype in the Pfister dataset [24]. Ubiquitination facilitates L1CAM protein degradation at the cell surface [25]. Additionally, over-expressed in ST_EPN_RELA was the gene encoding the stem cell marker and non-canonical Notch inhibitor, *DLK1* (F = 6.81, *p* = 2.96 × 10^−7^).

**Figure 10:**
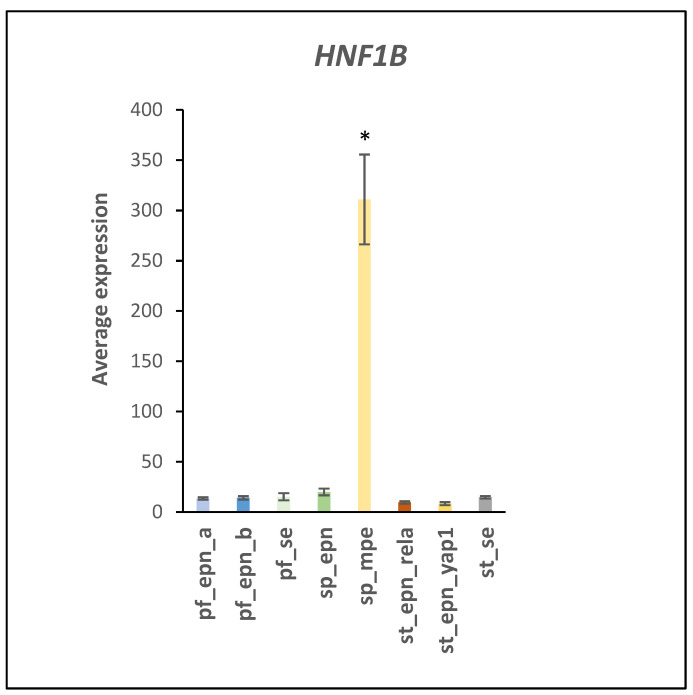
*HNF1B* expression in the SP-MPE subtype (by Anova, F = 151.68, *p* = 1.27 × 10^−76^) * by *t*-test significantly different from all other groups at *p* < 0.001. Dataset GSE64415.

*HNF1B* (Hepatic Nuclear Fact 1B, aka HNF1 homeobox B) is a gene located on chromosome 17 (17q12) and encodes a transcription factor that regulates Notch signaling [26] and facilitates stem cell differentiation [27]. In the EPN dataset, *HNF1B* expression was selectively elevated by approximately 20-fold (F = 151.68, *p* = 1.27 × 10^−76^) in the SP_MPE subgroup (Figure 10). Acetylation of HNF1B attenuates ubiquitin-proteasomal degradation [28].

**Figure 11:**
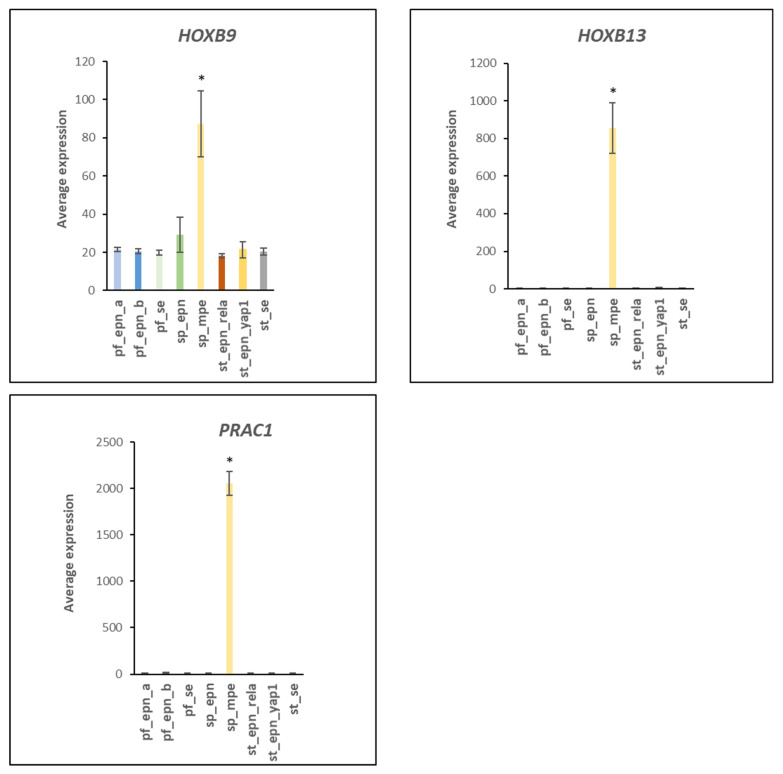
Gene expression in the homebox cluster of chromosome 17 (17q21.32) in the SPE_MPE subtype (by Anova, *HOXB9*, F = 25.31, *p* = 5.26 × 10^−25^; *HOXB13*, F = 160.28, *p* = 1.21 × 10^−78^; *PRAC1*, F = 953.53, *p* = 1.96 × 10^−150^). * for *HOXB9 p* < 0.01; for HOXB13, *p* < 0.0001; for *PRAC1*, *p* < 0.0001. Dataset GSE64415.

The R2 program identified three genes in the homeobox cluster of chromosome 17 (at 17q21.32) that were highly over-expressed selectively in SP_MPE. This included *HOXB9* (F = 25.80, *p* = 5.26 × 10^−25^), *HOXB13* (F = 160.28, *p* = 1.21 × 10^−78^; >150-fold), and *PRAC1* (F = 953.53, *p* = 1.96 × 10^−150^; >150 fold). Expression of the homeobox genes is shown in Figure 11 and may provide suitable new developmental markers of the SP_MPE subtype.

**Figure 12:**
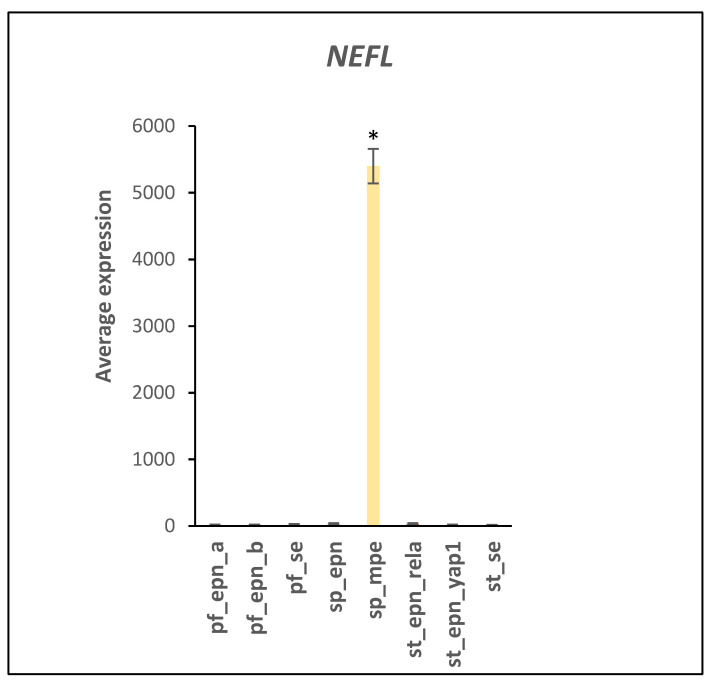
The *NEFL* gene is selectively overexpressed in SP_MPE (F = 1505.18, *p* = 7.05 × 10^−170^). * *p* < 0.001 by *t*-test, compared to all other groups. Dataset GSE64415.

Additonal homeobox genes, *HOXC13* (F = 32.41, *p* = 7.04 × 10^−30^) and *HOXA13* (F = 66.90, *p* = 4.13 × 10^−49^), located on the homebox clusters of chromosome 12 (12q13.13) and chromosome 7 (7p15.2), respectively, were also over-expressed in SP_MPE. The E3 ligases RNF20 and RNF40 have been shown to regulate many homeobox genes [29]. Expression of several other markers for the SP_MPE subtype were highly correlated with the expression of the homeobox genes. The *NEFL* (Neurofilament Light Chain) gene was found over-expressed in the SP_MPE subtype by more than 150-fold (F = 1505.18, *p* = 7.05 × 10^−170^) compared ot the other groups (Figure 12) and confirmed a previous report by Barton et al. [30]. The NEFL protein is a substrate of the ubiquitin ligase Trim2 [31,32]. Additionally, substantially increased in the SP_MPE subtype was the expression of the genes *EPHB2* (F = 64.970, *p* = 3.13 × 10^−48^) and *P2RX5* (F = 86.573, *p* = 3.35 × 10^−57^). The ubiquitin ligase SPSB4 regulates EPHB2 [33], while the UPS regulation of *P2RX5* has not been reported. In summary, the data suggest that *NEFL*, *EPHB2*, and *P2RX5* are useful genetic markers for the SP_MPE group and their proteins should be investigated further as to their potential as therapeutic targets.

**Figure 13:**
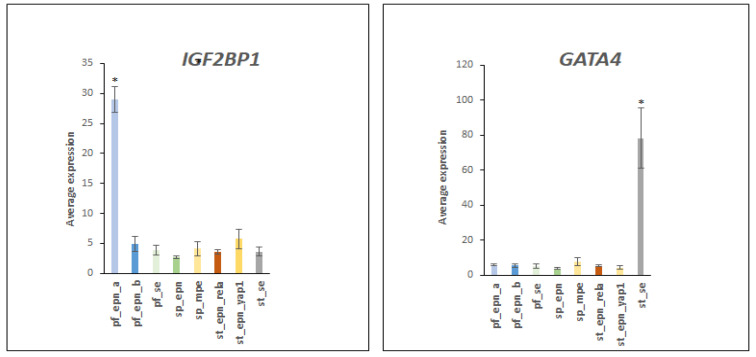
The *IGF2BP1* gene expression is selectively elevated in the PF_EPN_A subtype (F = 30.48, *p* = 1.66 × 10^−28^) and GATA4 expression is elevated in the ST_SE subtype (F = 57.00, *p* = 2.16 × 10^−44^). * significantly different from other groups by *t*-test *p* < 0.001 for *IGF2BP1* and *p* < 0.01 for *GATA4.* Dataset GSE64415.

Located on chromosome 17 at 17q21.32, IGF2BP1 is a regulator of RNA stability and over-expression of IGF2BP1 is associated with tumor progression and poor prognosis in a variety of cancers [34]. Of all EPN subtypes, *IGF2BP1* expression was selectively elevated in PF_EPN_A (F = 30.48, *p* = 1.66 × 10^−28^) (Figure 13). IGF2BP1 is a substrate of the E3 ligase adaptor FBXO45 [35] and potential cancer therapeutic target [36]. Elevated protein levels of the ubiquitin E3 ligase adaptor FBXO45 promote tumor progression by stimulating ubiquitination of IGF2BP1 in hepatic cancer [35]. Over-expression of IGF2BP1 is associated with poor prognosis in a variety of cancers [34]. FBXO45 is a therapeutic target in cancer [36] and IGF2BP1 may also be considered a potential therapeutic target in PF_EPN_A.

Expression of the transcription factor *GATA4* was selectively elevated in the ST_SE subgroup (Figure 13). GATA4 is regulated by sumolyation via the E3 ligase PIAS1 [37].

**Figure 14:**
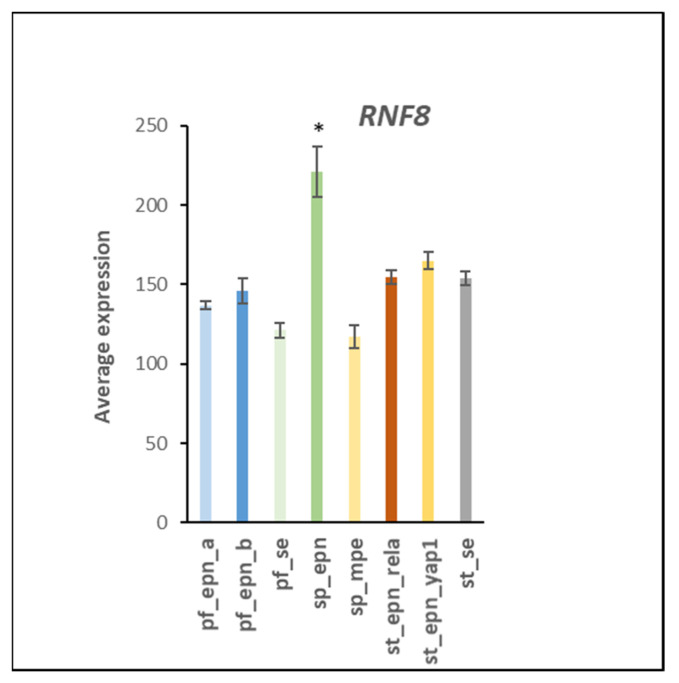
Elevated expression of E3 ligase RNF8 in the SP_EPN subtype (by Anova, F = 11.86, *p* = 1.28 × 10^−12^). * significantly different from all other groups by *t*-test *p* < 0.01. Dataset GSE64415.

The ubiquitin E3 ligase RNF8 plays an important role in response to DNA repair and interacts with E2 conjugase, UBE2N (UBC13), in response to DNA damage [38]. RNF8 is recruited to double strand breaks where it functions as the major E3 ligase for poly-ubiquitination of DNA damage related proteins [38,39]. In the Pfister EPN dataset, *RNF8* expression was elevated in the SP_EPN subtype (F = 11.86, *p* = 1.28 × 10^−12^), providing evidence for a transcriptional EPN subtype specific UPS gene signature on DNA damage response pathways (Figure 14).

### 3.6. Ubiquitin Ligase E3 Adaptor Gene Expression in EPN Subtypes

**Figure 15:**
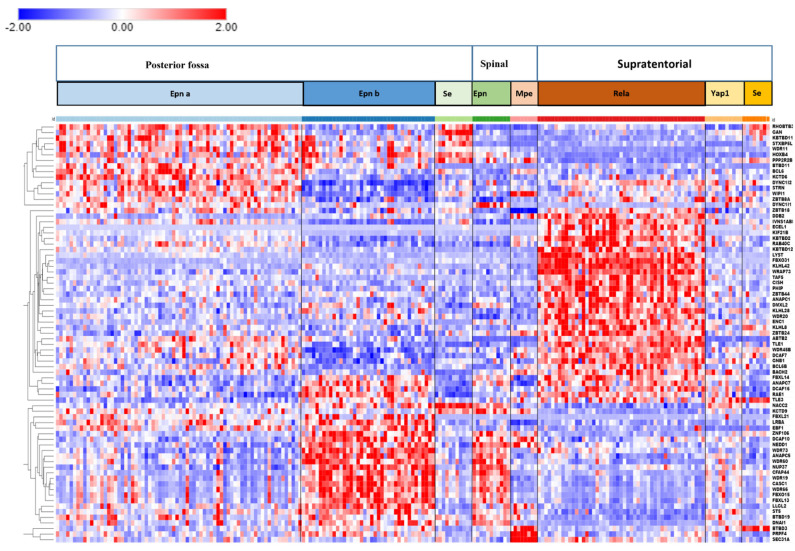
Cluster analysis of ubiquitin E3 ligase adaptor gene expression in EPN subtypes.

**Figure 16:**
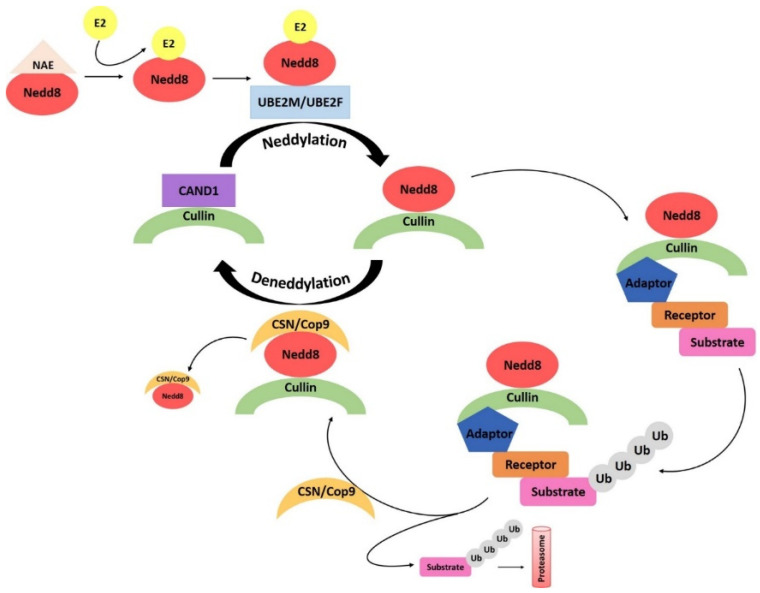
Steps in the Neddylation cycle: 1. Activation of Nedd8 by NAE, 2. Loading of Nedd8 to E2 UBE2M or UBE2F, 3. Displacement of CAND1 protein from Cullin Ring by Nedd8, 4. Neddylation of Cullin ring, 5. Assembly of Cullin Ring Ligase (CRL) with adaptor and substrate receptor, 6. Ubiquitination of target substrate, 7. CSN (Cop9 signalosome) binding to Nedd8, 8. Deneddylation, 9. Disassembly of CRL, 10. CAND1 binding to CRL.

Cluster analysis revealed that ubiquitin E3 ligase adaptor gene expression was distinctly different in EPN subtypes (Figure 15). Reactome *Pathway* analysis identified a significant association of ubiquitin E3 ligase adaptor genes depicted in the heatmap with the *Neddylation* pathway (12 genes) and genes associated with the *APC/c* (Anaphase promoting complex/cyclosome) E3 ligase complex (3 genes) (Table 6). Figure 16 illustrates the various steps in the neddylation cycle.

Order of genes displayed on the right side of the heatmap: RHOBTB3, GAN, KBTBD11, STXBP5L, WDR11, HOXB4, PPP2R2B, BTBD11, BCL6, KCTD6, DYNC1L2, STRN, WIPI1, ZBTB8A, DYNC1L1, ZBTB18, DDB2, IVNS1ABP, ECEL1, KIF21B, KBTBD2, RAB40C, KBTBD12, LYST, FBXO31, KLHL42, WRAP73, TAF5, CISH, PHIP, ZBTB44, ANAPC1, DMXL2, KLHL28, WDR20, ENC1, KLHL8, ZBTB24, ABTB2, TLE1, WDR45B, DCAF7, GNB1, BCL6B, BACH2, FBXL14, ANAPC7, DCAF16, RAE1, TLE3, NACC2, KCTD9, FBXL21, LRBA, EBF1, ZNF106, DCAF10, NEDD1, WDR73, ANAPC5, WDR60, NUP37, CFAP44, WDR19, CASC1, WDR66, FBXO15, FBXL13, LLGL2, ST5, BTBD19, DNA1, BTBD3, PRPF4, SEC31A.

**Figure 17:**
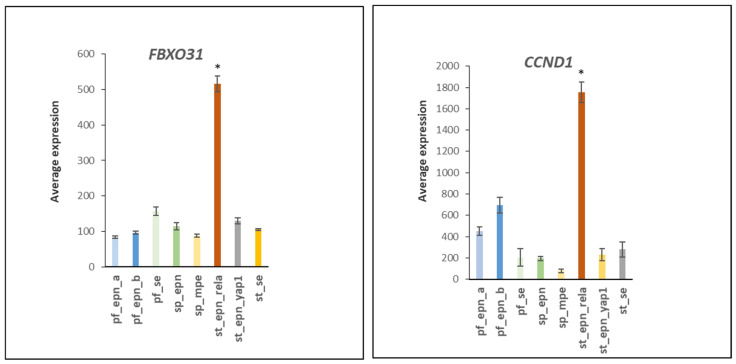
Elevated expression of the genes encoding ubiquitin E3 ligase adaptor *FBXO31* and its substrate cyclin D1 (*CCND1*) (by F = 153.74, *p* = 4.09 × 10^−77^; F = 51.39, *p* = 1.77 × 10^−41^) in the ST_EPN_RELA subtype. * significantly different from all other groups by *t*-test. Dataset GSE64415.

**Figure 18:**
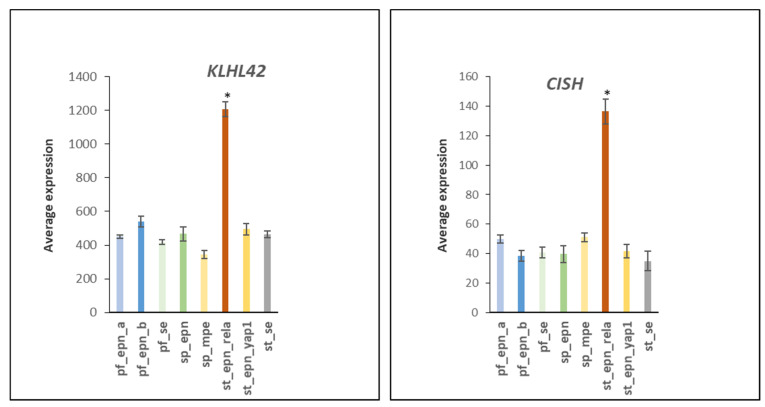
Elevated gene expression of *KLHL42* and *CISH* in ST_EPN_RELA (by Anova, F = 84.68, *p* = 1.75 × 10^−56^; F = 39.09, *p* = 2.55 × 10^−34^, respectively). * significantly different from all other groups by *t*-test *p* < 0.001. Dataset GSE64415.

**Figure 19:**
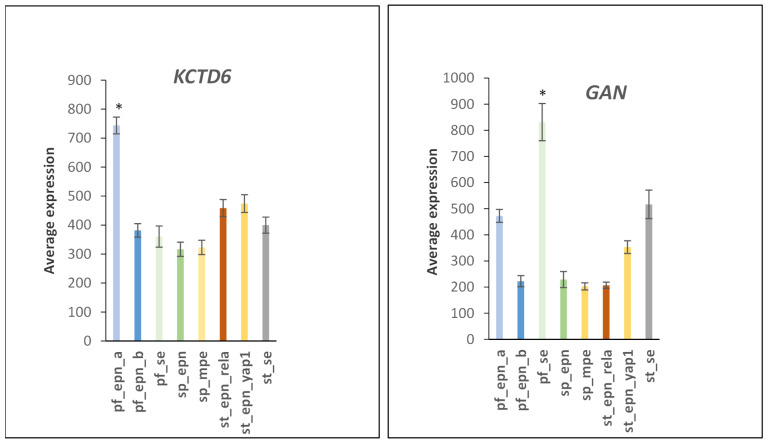
Elevated gene expression of *KCTD6* (pf_epn-a) and *GAN* (pf-se) (by Anova F = 21.73, *p* = 1.023–21 and F = 33.29, *p* = 1.71 × 10^−30^). * by *t*-test significantly different from other groups *p* < 0.001, for *KCTD6* and *p* < 0.01 for *GAN.* Dataset GSE64415.

Neddylation is essential for cell cycle progression [40,41,42] and dysfunction of neddylation regulation of E3 ligase complexes controlling the cell cycle contributes to one or more EPN subtypes. Neddylation reactions that were most significantly associated with the E3 ligase adaptor genes of Figure 15 are listed in Table 7. This includes *DCAF7*, *DCAF10*, *DCAF16*, *DDB2* genes, which encode proteins that serve as substrate recognition adaptors to CUL4 E3 ligase complexes. In particular, the *DCAF* genes encode substrate receptor proteins for CUL4-DDB1 E3 ligase complexes, whereas *DDB2* (Damage Specific DNA Binding Protein 2) encodes a substrate adaptor protein which is also a DNA damage sensor and part of the nucleotide excision repair (NER) pathway) [43,44,45]. Another group of neddylation associated genes encodes substrate recognition adaptors for Cullin 1 E3 ligase complexes (*FBXL13*, *FBXL14*, *FBX015*, *FBXO31*) and for Cullin Ring 4 and Cullin Ring 5 E3 ligase adaptors (*CISH*, *GAN*, *KCTD6* and *KLHL42*) (Table 7). Expression of the gene encoding the neddylation activating enzyme UBA3 (aka NAE2) was elevated in the ST_EPN_RELA subtype, suggesting enhanced neddylation as a relevant biological process in this EPN subtype. Elevated expression of several E3 adaptors associated with the Neddylation Reactome *Pathway* (Table 6) was identified in ST_EPN_RELA (*FBXO31*, *KLHL42*, and *CISH*), in PF_EPN_A (*KCTD6*), and in PF_SE (*GAN*) (Figure 17, Figure 18 and Figure 19).

Currently, little is known about the regulation and functional impact of neddylation of E3 ligases and adaptors in EPN. An increase in the expression of the protein SCCRO3 encoded by *DCUN1D3* (Defective in Neddylation 1D3) was shown to reduce neddylation of F-Box ubiquitin ligases (Cullin 1 ligases). SCCRO3 regulates neddylation by binding to CRL1 ligases and the protein CAND1 to block the nuclear translocation of E3 ligases [46], which contributes to cell cycle progression upon UV damage [47]. In the EPN dataset, expression of the *DCUN1D3* gene was elevated in the SP_MPE subgroup (F = 11.62, *p* = 2.22 × 10^−12^), suggesting a dysregulation of neddylation of E3 ligases by SCCRO3 protein in this EPN subtype.

The several-fold elevated expression of *FBXO31* and its substrate *CCND1* selectively in ST_EPN_RELA (F = 51.39, *p* = 1.77 × 10^−41^) may contribute to cell cycle dysregulation in ST_EPN_RELA (Figure 17). The E3 ligase adaptor protein FBXO31 was identified as a tumor suppressor in breast cancer [48] and was shown to stimulate cell proliferation, invasion and metastasis in lung cancer [49]. FBXO31 binds to the cell cycle regulator cyclin D1 (CCND1) to cause the ubiquitination and degradation of CCND1 [50]. Over-expression of FBXO31 after DNA damage results in cell cycle arrest in G1 [51].

Expression of the *KLHL42* (aka *KLHDC5*) adaptor of an E3 ligase complex necessary for normal mitosis was elevated >2-fold in ST_EPN_RELA (F = 84.68, *p* = 1.75 × 10^−56^) [52]. Similar findings were observed with the E3 adaptor gene *CISH* (F = 39.09, *p* = 2.55 × 10^−34^), which encodes a member of the SOCS (Suppressor of Cytokine Signaling) family of proteins (Figure 18).

Gene expression of the ubiquitin E3 adaptor *KCTD6* was highly elevated in PF_EPN_A compared to other EPN subtypes (F = 21.73, *p* = 1.02 × 10^−21^), while expression of *GAN* was highly elevated in PF_SE (F = 33.29, *p* = 1.71 × 10^−30^) (Figure 19).

### 3.7. Ubiquitin E3 Ligase Adaptors and Viral Markers

**Figure 20:**
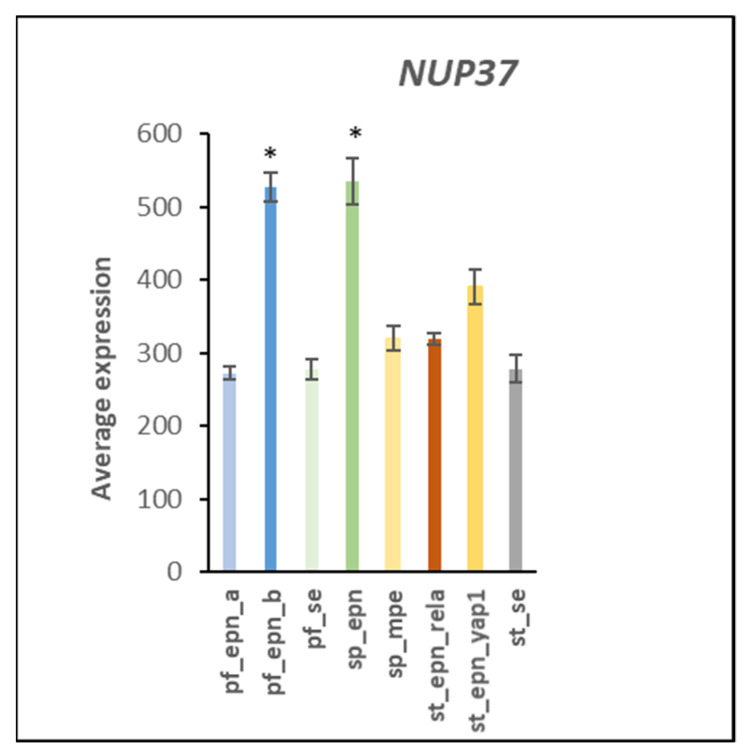
Differential expression of genes encoding E3 ligase adaptors related to the nuclear pore gene *NUP37* (by Anova, F = 43.18, *p* = 7.89 × 10^−37^). * PF_EPN_B and SP_EPN means are significantly higher than the other groups by *t*-test *p* < 0.01. Dataset GSE64415.

**Figure 21:**
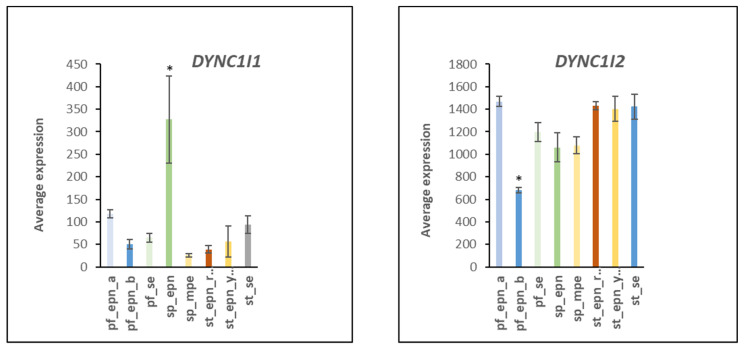
Differential expression of genes encoding E3 ligase adaptors associated with the dynein motor complex (by Anova, F = 13.835, *p* = 1.38 × 10^−14^; F = 27.89, *p* = 1.38 × 10^−14^). * by *t*-test DYNC1I1 expression is elevated in the SP_EPN group *p* < 0.05, while *DYNC1I2* expression is significantly lower in the PF-EPN-B group *p* < 0.001, compared to each of the other groups. Dataset GSE64415.

A role for human cytomegalovirus (HCMV) in a variety of tumors has recently been proposed due to the widespread detection of this virus in human cancers [53]. Table 7 summarizes evidence for an involvement of several different ubiquitin E3 ligase adaptors (DYNC1I1, DYNC1I2, NUP37, RAE1) associated with HCMV infection. Some viruses can facilitate the transport of their genome into the nucleus through nuclear pore complexes [54,55] and the Reactome program identified the *HCMV nuclear pore docking* pathway in the EPN dataset. NUP37 is a marker for various cancers and is required for the assembly of the nuclear pore complex and mitosis [56,57]. In the EPN dataset, *NUP37* expression was elevated in the PF_EPN_B and SP_EPN subtypes (Figure 20). Expression of *DYNC1I1* was elevated in SP_EPN and expression of *DYNC1I2* was depressed in PF_EPN_B (Figure 21). *DYNC1I1* and *DYNC1I2* encode E3 ligase adaptor proteins that are part of the Dynein transport system along microtubules [58]. Dynein has been shown to interact with components of the nuclear pore.

### 3.8. Anaphase Promoting Complex/Cyclosome (APC/c) E3 Ligase Adaptors

Three genes encoding for E3 ligase adaptors were identified by the Reactome program as genes in the heatmap of Figure 15 that encode for components of the APC/c ubiquitin ligase complex, including *ANAPC1*, *ANAPC5*, and *ANAPC7*. These genes are associated with the degradation of cell cycle proteins Cyclin A, Cyclin B, and securin. We suggest a role for the APC/c complex in EPN oncogenesis and implicate dysregulation of the APC/c complex as a contributor towards a subtype dependent EPN pathogenesis. This is supported by data showing that UBCH10 (UBE2C) initiates ubiquitination of the APC/c ubiquitin E3 ligase complex [14,15,16] and that the expression of *UBE2C*, *UBE2S* and *ANAPC1*, are elevated in the ST_EPN_RELA subtype (Figure 3).

## 4. Discussion

Our analysis of the EPN data sets identified several genes encoding ubiquitin E1 activators, E2 conjugases, and E3 ligases and adaptors of the UPS pathway that were highly differentially expressed in specific EPN subtypes. GO and *Reactome* analyses provided valuable information on a diverse set of probable biological pathways supported by the UPS genes in EPN subtypes.

### 4.1. Setting the UPS Stage: Ubiquitin E1 Activator

Forming a heterodimer with NAE1 that activates and transfers NEDD8 to ubiquitin conjugases (Figure 16), the ubiquitin activator UBA3 (aka Neddylation activating enzyme NAE2) was significantly elevated in the ST_EPN_RELA subtype (Figure 1). This may suggest a particular involvement of UBA3/NAE2 gene product in enhanced activation of NEDD8 in the ST_EPN_RELA subtype. The notion that neddylation is a major pathway distinguishing the EPN subtypes was further supported by our analysis of ubiquitin ligase adaptors (Table 6 and Table 7), which were also associated with an aberrant neddylation pathway in some EPN subtypes. In the order of significance, high gene expression of *UBA1*, *UBA6*, and *UBA3/NAE2* were associated with significantly shorter survival times in the French glioma dataset (chi = 26.79, *p* = 2.3 × 10^−7^, chi = 21.04, *p* = 4.5 × 10^−6^, chi = 8.79, *p* = 3.0 × 10^−3^, respectively). TAK-243 is a small molecular inhibitor of the proteins UBA1, UBA6 and NAE, used for cancer treatment in pre-clinical animal models [59,60]. TAK-243 is also undergoing Phase 1 clinical trials in patients with refractory acute myeloid leukemia (AML) to block UBA1, UBA6, and NAE targeted proteins involved in tumor cell division (ClinicalTrials.gov Identifier: NCT03816319). A neddylation inhibitor MLN4924 (aka Pevonedistat) that interacts with UBA3/NAE2 [61] blocks activation of many cullin ring ligases. Pevonedistat has been shown to have therapeutic effects in several, but not all, cancer clinical trials [62,63,64], and is undergoing clinical trials for glioblastoma [65,66]. The E2 conjugase protein UBE2Z (aka UBA6-specific enzyme) is a substrate of the UBA6 E1 activator. The expression of *UBE2Z* gene was elevated in the SP-MPE subtype relative to the other EPN subtypes (F = 28.73, *p* = 3.14 × 10^−27^), highlighting UBE2Z as a possible specific therapeutic target for the SP_MPE subytpe.

### 4.2. Immune Recognition and Inflammation

The UPS plays a critical role in regulation of the immune response [67] and is required to process cytoplasmic proteins into MHC class I antigenic peptides prior to surface presentation on antigen presenting cells for recognition by T cells [68,69]. The proteasome responds to immunological challenges with specific modifications that result in the formation of an immunoproteasome, in which the 3 catalytic subunits, β1, β2, and β7 have been replaced by catalytic immuno-subunits LMP2, MECL-2, and LMP7 under the influence of interferon gamma (IFN-γ) [68]. A major *Reactome* pathway associated with differential expression of E2 ubiquitin conjugase genes (other than the E2 category itself) was *“Antigen processing: ubiquitination and proteasome degradation”* (Table 2). Both ubiquitin E2 conjugases, UBE2H (aka UBC8) and UBE2L6 (important in ISGylation), are interferon-inducible ubiquitin E2 conjugases and may be involved in the process of antigen presentation and EPN differentiation (Table 2) [70,71,72]. The UPS can affect antigen presentation to attenuate immune recognition and prevent destruction by reactive humoral and cellular immune responses [73]. Coinciding with our finding of differential expression of genes encoding ubiquitin E2 conjugases in this pathway (Table 2), several ubiquitin E3 ligases in EPN subtypes were also associated with the Reactome *Pathway* of *Antigen processing: ubiquitination & proteasome degradation* (Table 4). Ubiquitination of the MHC class I heavy chain is required for translocation into the cytosol [74,75], where antigenic peptides are produced by the 26S proteasome. Membrane-anchored E3 ligases of the Modulator of Immune Recognition (MIR) family of E3 ligases affect antigen presentation by regulating lysosomal degradation of MHC class I antigens [76]. Multiple E3 ubiquitin ligase genes of the TRIM family are located in the MHC Class I region of chromosome 6 [77]. This includes *TRIM27*, a gene included in the heatmap of differentially expressed E3 ligases in EPN (Figure 5). The network analysis of Figure 6 illustrates the interaction of other E3 ligases on *TRIM27* expression. The expression is related to inflammatory disesases and viral infections [77]. While some TRIM proteins have been associated with cancer [78], we could find no previous reports relating *TRIM27* to EPN tumorigenesis.

Several lines of evidence in the UPS data suggested an association between cellular responses to viral infection and inflammation in EPN and warrant further investigations into viral contributions to EPN subtypes. Primarily with the PF_EPN_B subtype, we identified the Reactome Pathway “*HCMV nuclear pore docking pathway*” associated with differential expression of genes encoding E3 adaptors that are part of the dynein motor complex (Table 7). Interferon gamma (IFNγ) stimulates many genes associated with the MHC class 1 immune response [79]. Induction of IFNγ promotes immunoproteasome functionalities to facilitate the degradation of endogenous proteins into antigenic peptides for binding to MHC class I proteins prior to translocation to the cell membrane and presentation of these MHC class I-peptide complexes to CD8 T cells [68]. The induction of IFNβ- and IFNγ-inducible genes is regulated by the ubiquitin E3 ligase PIAS1 (Protein Inhibitor of activated Stats 1) [80]. Expression changes in UPS genes identified in both ST_EPN_RELA and PF_EPN_B included several differentially expressed UPS genes associated with interferon gamma signaling (*MID1*, *PIAS1*, *TRIM2*, *TRIM22*, *TRIM45*) that may promote a proinflammatory milieu similar to that observed upon viral infections (Table 4). INFγ stimulates the transcription of *PSMB8* (LMP7), *PSMB9* (LMP2), *PSMB10* (LMP10) immunoproteasome subunit genes, proteasome activator genes *PSME1* (PA28α) and *PSME2* (PA28β), the transporters associated with antigen processing, *TAP1* and *TAP2*, and the MHC class 1 genes, *HLA.A*, *HLA.B*, and *HLA.C* [79]. A number of TRIM E3 ligase proteins, including TRIM2 and TRIM22, were specifically down-regulated in PF-EPN_A but not PF-EPB_B. TRM2 and TRIM22 play a role in antiviral immunity [81,82,83] and are either induced and/or activated by interferon gamma [84]. The UPS also appears to have functions associated with early cellular warning systems of viral infections. The *IFIH1* gene product, melanoma differentiation associated gene-5 (MDA5), and the DDX58 gene product retinoic acid inducible gene-I (RIG-I) are intracellular RNA sensors and part of an elaborate cellular defense system against viral challenges that stimulates type 1 interferon production [85]. This includes endoplasmic reticulum (ER)-associated virus inhibitory protein RSAD2, radical S-adenosyl methionine domain containing 2, which is a downstream target of pattern RNA sensing Toll-like receptor (TLR) 7, and also selectively increased in PF-EPB_B. These data suggest that the expression changes in UPS genes identified in both ST_EPN_RELA and PF_EPN_B may promote a proinflammatory milieu similar to that observed upon viral infection.

### 4.3. UPS and NOTCH Signaling

HDAC4 protein stimulates IFNγ (Type 2 interferon signaling) [86] and HDAC inhibition is considered a potential therapeutic strategy in pediatric brain cancers [87,88]. Although the effect of HDAC inhibitors on acetylation-dependent and acetylation-independent mechanisms is well documented, we were unable to identify studies that determined the effect of HDAC inhibitors on ubiquitin E3 ligase/SUMOylation activity of HDAC4. The regulation of *HDAC4* gene expression is incompletely understood but emerging evidence suggests an involvement of post-translational protein modifications, because neddylation deficiency was reported to enhance the *HDAC4* mRNA and protein expression in myoblasts [88]. Importantly, HDAC enzymes can inhibit Notch signaling but a role of HDAC4 ubiquitin E3 ligase activity in this signaling process remains largely unclear [89,90]. Our Reactome network analysis identified a relationship of differentially expressed E3 ligases and Notch signaling pathway (Figure 6). Many Notch target genes have been identified that show cell-type specific expression [91] and contribute to unique roles in Notch directed context-dependent development and differentiation in different cell types [21]. Notch nuclear signaling via its intracellular domain (NICD) stimulates transcription of developmental genes linked to cancer [91,92,93]. Ubiquitin E3 ligases continue to emerge as important post-translational modulators of NOTCH signaling. Deltex E3 ligase proteins have key functions in the cleavage and endocytosis of the Notch receptor and the release of its NICD domain [94,95,96]. These Deltex E3 ligase proteins are encoded by the Deltex genes *DTX1*, *DTX2*, *DTX3*, *DTX3L*, and *DTX4*. SUMOylation was reported to promote enhanced nuclear localization of NICD but decrease expression of some Notch target genes during in vitro cell stress [97]. HDAC4 is attracted to the NICD transcriptional complex during stress-induced SUMOylation and has SUMO E3 ligase activity [18,19]. Phosphorylation of NICD followed by ubiquitination by an E3 ligase complex leads to NICD degradation [91]. The ubiquitin ligase adaptor for this complex, FBXW7 (aka FBW7) [98], is one of the most commonly dysregulated UPS proteins in human cancers [91,99]. Inhibition of FBXW7 would be expected to attenuate the degradation of NICD protein. Our analysis implicate over-expression of a subset of Notch target genes as part of an oncogenic molecular signature of ST_EPN_RELA. In addition, in supratentorial (ST) EPN, NOTCH1 expression was associated with cancer stem cell (CSC) markers, *VEGFA* and *L1CAM*, and the ST_EPN_RELA subtype showed the activation of selected key members of the Notch signaling pathway (NOTCH1, JAG1, JAG2, HES4) [22]. The *L1CAM* gene was strong marker for the ST_EPN_RELA subtype (Figure 9). Ubiquitination of full length L1CAM protein regulates its lysosomal degradation and intracellular trafficking [25], while SUMOylation of a smaller 70 kDa intracellular fragment is required for nuclear translocation [100]. Lutz et al. [100] found that the L1CAM 70 kDa fragment was generated in the mouse primarily before postnatal Day 3.

### 4.4. The Anaphase Promoting Complex/Cyclosome (APC/c)

The role of the APC/c complex in cancer development and progression has been reviewed by VanGendersen and colleagues [101]. Dysregulated gene transcription of E2 conjugases and E3 ligase adaptors of the APC/c complex was detected in the ST-EPN-RELA subgroup. The APC/c complex is an ubiquitin E3 ligase complex that regulates mitotic exit by stimulating the transition from metaphase to anaphase by ubiquitination and proteasomal degradation of securin and cyclin B [102]. UBE2C and UBE2S ubiquitinate the APC/c ubiquitin ligase complex, facilitating the conversion of APC/c -Cdc20 to APC/c-Cdh1 which is required for the transition to anaphase in the cell cycle [14,15,16]. Increased expression of *UBE2C* (F = 9.92, *p* = 1.28 × 10^−10^) observed in ST_EPN_RELA of the Pfister dataset (Figure 3) has been associated with aggressive progression and poor outcome of malignant glioma [103]. Over-expression of UBE2C protein stimulates cell proliferation [104,105], may act as an important proto-oncogene [105,106] and is found over-expressed in several cancers [105,106,107,108,109,110,111,112,113]. Donato and colleagues proposed suppression of UBE2C (UBCH10) as a potential therapy for astrocytic brain tumors [114]. UBE2C may also be a therapeutic target in breast cancer [115] and ovarian cancer [112]. Increased expression of *UBE2C* in PF-EPN-RELA (Figure 3) suggests that this supratentorial EPN may also benefit from UBE2C targeted therapies. ST-EPN_RELA also showed increased expression of *UBE2S* (F = 16.00, *p* = 1.19 × 10^−16^) (Figure 3). The UBE2S protein was recently described as an oncogene with potential as a therapeutic target in various cancers [116]. The elevated expression of *UBE2S* may qualify as a genetic marker for ST-EPN-RELA and its gene product may present a promising therapeutic target in EPN. Additionally, differentially expressed in EPN subtypes were the ubiquitin ligase adaptors of the APC/c ubiquitin ligase complex, ANAPC1, ANAPC5, and ANAPC7, with functions in mitotic exit (Table 7). The gene coding for a protein of *ANAPC1* was up-regulated (F = 22.95, *p* = 1.0 × 10^−22^) in the ST-EPN-RELA subtype relative to the other EPN subtypes, while expression of *ANAPC5* was depressed relative to the other EPN subtypes (F = 11.26, *p* = 5.22 × 10^−12^). The data suggest dysregulation of the APC/c complex in ST_EPN_RELA and possibly in one or more additional EPN subtypes.

### 4.5. Neddylation

A main Reactome *Pathway* over-represented by ubiquitin ligase adaptor genes in EPN subtypes was the Neddylation pathway (Figure 15; Table 6). The Neddylation cycle (Figure 16) includes various steps and contributes to oncogenesis by affecting cell cycle regulation, autophagy, DNA repair, and genomic stability [42]. Our analysis suggests that transcription of all three levels of the UPS pathway, E1 activation, E2 conjugation, and E3 ubiquitination, are modulated in a subtype specific manner. In EPN, transcriptional regulation of genes associated with the Neddylation pathway appeared to contribute to EPN oncogenesis in a subtype-specific manner and these genes may serve as genetic markers for specific EPN subtypes. The use of specific inhibitors for Neddylation components, such as UBE2M inhibitors [42] or MLN4924, which induced cell cycle arrest in human osteosarcoma [117], may be attractive new avenues for the treatment of the ST_EPN_RELA subtype but the development of more specific inhibitors of CRLs is warranted to benefit EPN patients. The Reactome *reactions* of the E3 ligase adaptor genes in Table 7 provided further information on differential regulation of neddylation in the EPN subtypes.

### 4.6. Linking UPS with Genomic Stability and DNA Repair in EPN Subtypes

Ubiquitin E2 conjugase genes were expressed greastest in ST_EPN_RELA and included *UBE2T*, *UBE2J2*, *UBE4B*, *UBE2Q1*, *UBE2V2* and *UBE2I*. Their role in EPN is currently largely unknown. UBE2T is part of the Fanconi Anemia (FA) core complex and required for the activation of the FA pathway in DNA repair [118]. We could not find information in the literature regarding *UBE2J2* expression in EPN. However, Chen et al. [119] reported higher expression of UBE2J2 protein in hepatocellular carcinoma compared to normal liver tissue, which was associated with increased epithelial to mesenchymal transition (EMT) in vitro, whereas *UBE2J2* gene knockdown promoted EGFR expression [119]. UBE4B regulates the degradation of the tumor suppressor p53 in breast cancer [120] and UBE2Q1 has been proposed as a marker for high-grade serous ovarian cancer [121]. Ubiquitin E2 conjugases affecting ubiquitination and SUMOylation of Proliferating Cell Nuclear Antigen (PCNA) were associated with ST-EPN-RELA subtype and affect the complex functions of PCNA at DNA replication forks and in DNA repair [122,123]. The ubiquitin E2 conjugases UBE2N and UBE2V2 contribute to ubiquitination of PCNA to allow the continuation of the DNA replication process across unrestored damaged DNA sites [124,125]. Expression of both *UBE2V2* and *UBE2I* (aka SUMO Conjugating Enzyme, *UBC9*) were elevated in the ST_EPN_RELA compared to the other subtypes. UBE2I contributes to SUMOyation of PCNA and is a proposed therapeutic target in colon cancer [124,126,127] and the SUMO conjugating activity of UBE2I also contributes to DNA repair [128]. *RNF8* expression was elevated in SP_EPN (Figure 14). The E3 ligase RNF8 interacts with the E2 conjugase UBE2N during double stranded DNA repair [38,39]. Our analysis suggests a possible misregulation of double stranded DNA damage and repair in SPN_EPN.

### 4.7. Developmental Factors in EPN Subtypes

We identified over-expression of several homebox genes specifically in SP_MPE. The expression of *HOXB9*, *HOXB13*, and PRAC1 and PRAC3 genes are all located in the cytogenetic band 17q21.32 as part of the human HOX gene region (Figure 11). The over-expression of HOX genes in SP-MPE has been previously reported, along with data showing evidence for the over-expression of the HOXB13 protein by immunohistochemistry [30]. The gene for the ubiquitin conjugase *UBE2Z* is also located at 17q21.32, a finding not previously noted. Since HOX genes outside of chromosome 17 are also over-expressed in SP_MPE, the data suggest that a single factor stimulates selected HOX gene expression on different chromosomes. UBE2I is likely the ubiquitin conjugase transferring SUMO to PIAS1 [129,130,131]. UBE2I and the E3 ligases RNF20 and RNF40 have been implicated in the regulation of homeobox gene expression and this involves histones H2B residue lysine 120 mono-ubiquitination and subsequent methylation at H3 at lysines 4 and 79 [29]. Genetic markers correlated with the increased HOX genes in SP_MPE included the *NEFL* gene whose expression is regulated by the UBE2D1 (UBCH5a) dependent ubiquitin E3 ligase TRIM2 [31]. The expression of *GATA4* was elevated in ST_SE (Figure 13). In cardiac tissues, GATA4 activity was demonstrated to be stimulated upon SUMOylation by the E3 ligase PIAS1 [37]. GATA4 has been found to be a tumor suppressor in astrocytomas [132]. Our analysis suggest GATA4 could be a marker for ST_SE. PIAS1 was elevated in PF_EPN_B (F = 20.17, *p* = 2.14 × 10^−20^).

### 4.8. Summary Table

A summary table of the major over-expressed pathways associated with the UPS components, E1 activators, E2 conjugases, and E2 ligases and adaptors are shown in Table 8. The table shows that more than one UPS component are associated with antigen processing, neddylation, and cell cycle pathways.

### 4.9. Current Targeting Approach for the UPS Pathway

Small molecular inhibitors have been reported that target ubiquitin E1 activators, the E2 conjugase UBE2N, the E3 ligases CRBN and MDM2, the E3 ligase adaptors SKP2 and BTRC, and the deubiquitinases USP2, USP7, and USP14 [134]. These molecules are in various stages of preclinical or clinical trials.

A neddylation inhibitor MLN4924 is undergoing clinical trials as an anti-cancer drug for solid tumors and hematologic malignancies [135].

PROTAC (Proteolyis TArgeting Chimera) technology is a way of engineering small molecular inhibitors which use the cells’ own E3 ligases to target substrates of interest [136]. This technology has great potential for targeting tumor proteins for degradation by the proteasome. A small molecule PROTAC was reported to inhibit MDM2-p53 interaction [137]. At least 12 PROTACs that target cancer have entered clinical trials since 2019 [138]. A PROTAC designed to target the CDC20-APC/c complex and inhibit mitotic progression of breast cancer cells in vitro was reported in 2019 [139]. PROTAC may also be a promising therapeutic strategy to target ependymoma with elevated expression of CDC20, including the ST_EPN_RELA and PF_EPN_A subgroups. Furthermore, our work has identified several other over-expressed proteins in selected subtypes of ependymoma which may provide attractive targets for future therapeutic applications using PROTAC technology. 5. Conclusions

Our analysis of the Pfister EPN dataset identified significant differential transcription of specific genes for ubiquitin E1, E2, and E3 components of the UPS system. High expression of the E2 conjugase UBE2C is associated with several aggressive cancers, including glioblastoma [103,106] and we can now include the ST_EPN_RELA subtype as well. UBE2C and UBE2S ubiquitinate substrates sequentially and may serve as suitable new therapeutic targets in ST_EPN_RELA. Another E2 conjugase, UBE2Z, may be serve as EPN subtype-specific therapeutic target for SP_MPE. The E2 conjugase UBE2M, a Nedd8 conjugating enzyme, emerged as a potential target for the inhibition of neddylation of E3 cullin ligases, which depend on this neddylating enzyme. The SUMOylation conjugating enzyme, UBE2I, which has been proposed as a therapeutic target in other cancers, may also be considered a valuable therapeutic target in ST-EPN-RELA. Reactome analysis of differentially expressed genes encoding ubiquitin E3 ligases and their adaptors in EPN identified proteins regulating MHC class 1 antigen presentation. SUMOylation may emerge as an important process regulating differential expression of E3 ligase proteins in EPN subtypes. Reactome analysis of ubiquitin E3 substrate adaptors implicated the Neddylation pathway and the APC/c complex as regulators of cell cycle progression in the pathology of EPN. Viral interference with ubiquitin ligase complexes associated with the nuclear pore may contribute to EPN pathogenesis at least in some EPN subtypes but the precise role of viruses, including HCMV, in EPN pathogenesis has yet to be elucidated. A great deal of research is still needed to validate the role of UPS genes in EPN pathogenesis. Our analysis provides a first unique insight into the proteins encoded by these genes and their substrates that promise a new attractive repertoire of biomarkers and potential therapeutic targets for EPN subtype-specific treatment of ependymoma patients.

## Figures and Tables

**Table 1:** Age and ependymoma cases * in subtypes of the Pfister dataset. Dataset GSE64415.

Age/Subtype	0–10	11–20	21–30	31–40	41–50	51–60	61–70	Total
PF-EPN-A	54	9	0	1	0	1	0	65
PF-EPN-B	0	9	12	7	5	3	0	36
PF-SE	1	0	0	1	1	5	3	11
SP-EPN	1	1	1	2	4	1	0	10
SP-MPE	1	2	2	1	0	2	0	8
ST-EPN-RELA	30	7	2	0	1	0	0	40
ST-EPN-YAP1	5	0	1	0	0	1	0	7
ST-SE	0	1	2	2	2	0	1	8

* age of diagnosis data available in 185/209 cases.

**Table 2:** Reactome *Pathways* * associated with differentially expressed genes encoding for E2 conjugases.

Gene Classification	Number of Reactome *Pathways*	Number of E2 Genes Associated with This Pathway	Genes	*p* Value for Group
Synthesis of active ubiquitin: roles of E1 and E2 enzymes	1	17	*UBE2A*, *UBE2B*, *UBE2C*, *UBE2D1*, *UBE2D2*, *UBE2E1*, *UBE2E3*, *UBE2G1*, *UBE2G2*, *UBE2H*, *UBE2K*, *UBE2L3*, *UBE2Q2*, *UBE2S*, *UBE2T*, *UBE2W*, *UBE2Z*	2.84 × 10^−39^
Protein ubiqutination	3	20	*UBE2A*, *UBE2B*, *UBE2C*, *UBE2D1*, *UBE2D2*, *UBE2E1*, *UBE2E3*, *UBE2G1*, *UBE2G2*, *UBE2H*, *UBE2J2*, *UBE2K*, *UBE2L3*, *UBE2N*, *UBE2Q2*, *UBE2S*, *UBE2T*, *UBE2V2*, *UBE2W*, *UBE2Z*	2.84 × 10^−39^
Antigen processing: ubiquitination and proteasome degradation	2	25	*UBE2E1*, *UBE2E2*, *UBE2G1*, *UBE2A*, *UBE2G2*, *UBE2C*, *UBE2B*, *UBE2E3*, *UBE2H*, *UBE2K*, *UBE2Q1*, *UBE2Q2*, *UBE2D1*, *UBE2L6*, *UBE3B*, *UBE2D4*, *UBE2D2*, *UBE2V2*, *UBE2N*, *UBE2S*, *UBE2W*, *UBE2J2*, *UBE2Z*, *UBE2J1*, *UBE2L3*	1.40 × 10^−33^
DDX58/IFIH1 and induction of interferon-alpha/beta	2	4	*UBE2D1*, *UBE2D2*, *UBE2K*, *UBE2L6*	2.63 × 10^−6^
IKK complex and RIP1	2	3	*UBE2D1*, *UBE2D2*, *UBE2N*	2.63 × 10^−6^
APC/C:Cdc20 conversion	22	4	*UBE2C*, *UBE2S*, *UBE2D1*, *UBE2E1*	3.27 × 10^−4^
Formation of incision complex in Global Genome nucleotide excision repair (GG-NER)	1	3	*UBE2I*, *UBE2N*, *UBE2V2*	1.41 × 10^−4^
ISG15 antiviral mechanism	1	3	*UBE2E1*, *UBE2L6*, *UBE2N*	6.52 × 10^−4^

* Reactome pathway was selected when a minimal number of three genes of all genes associated with the pathway were selected and represented at least 4% of the total genes in the pathway.

**Table 3:** Main Reactome *Reactions* over-represented by differentially expressed E2 conjugase genes.

Gene Classification	Reactome *Reactions*	Number of Genes	Genes	*p* for Group
Transfer of ubiquitin	4	25	*UBE2A*, *UBE2B*, *UBE2C*, *UBE2D1*, *UBE2D2*, *UBE2D4*, *UBE2E1*, *UBE2E2*, *UBE2E3*, *UBE2G1*, *UBE2G2*, *UBE2H*, *UBE2J1*, *UBE2J2*, *UBE2K*, *UBE2L3*, *UBE2L6*, *UBE2N*, *UBE2Q1*, *UBE2Q2*, *UBE2S*, *UBE2V2*, *UBE2W*, *UBE2Z*, *UBE3B*	2.89 × 10^−39^
UBA1 conjugates ubiquitin to cytosolic E2	1	11	*UBE2C*, *UBE2D1*, *UBE2D2*, *UBE2E3*, *UBE2G1*, *UBE2G2*, *UBE2H*, *UBE2K*, *UBE2L3*, *UBE2Q2*, *UBE2S*	3.90 × 10^−28^
UBA6 conjugates ubiquitin to cytosolic E2	1	7	*UBE2D1*, *UBE2D2*, *UBE2E3*, *UBE2G2*, *UBE2L3*, *UBE2S*, *UBE2Z*	3.90 × 10^−28^
UBA1 conjugates ubiquitin to nuclear E2	1	10	*UBE2A*, *UBE2B*, *UBE2C*, *UBE2D2*, *UBE2E1*, *UBE2E3*, *UBE2L3*, *UBE2S*, *UBE2T*, *UBE2W*	3.22 × 10^−23^
APC/c * related reactions	37	4	*UBE2C*, *UBE2S*, *UBE2D1*, *UBE2E1*	7.02 × 10^−10^
PCNA related reactions	5	3	*UBE2B (aka RAD6B), UBE2N*, *UBE2V2*	2.09 × 10^−5^

* APC/c-anaphase promoting complex/cyclosome.

**Table 4:** Main Reactome *pathways* over-represented in list of E3 ligase genes of Figure 5.

Reactome Term	No. of Reactome *Pathways*	No. of E3 Ligase Genes in Pathway	Genes	*p* for Pathway
Antigen processing: Ubiquitination & proteasome degradation	1	13	*AREL1*, *DZIP3*, *HACE1*, *HECW2*, *HERC6*, *PJA1*, *RNF114*, *RNF19A*, *RNF34*, *SMURF2*, *TRIM71*, *TRIM9*, *UBE3D*	4.94 × 10^−10^
Interferon gamma signaling	1	5	*MID1*, *PIAS1*, *TRIM2*, *TRIM22*, *TRIM45*	4.78 × 10^−5^
SUMOlyation of intracellular receptors	1	3	*HDAC4*, *PIAS1*, *PPARG*	3.03 × 10^−4^
SUMOlyation of Ubiquitinylation proteins	1	3	*MDM2*, *PIAS1*, *TRIM27*	6.14 × 10^−4^
Signaling by Notch	1	3	*DTX1*, *DTX4*, *HDAC4*	4.22 × 10^−3^

**Table 5:** Main interactome *Reactions* over-represented by E3 ligase genes of Figure 5.

Reaction Term	No. of Reactome *Pathways*	No. of E3 Ligase Genes in Pathway	Genes	Group *p* Value
1.Interaction of E3, E2-Ub complex and substrate 2.Transfer of UB from E2 to substrate 3.Polyubiquitination of substrate 4. Release of E3 from substrate	4	13	*AREL1*, *DZIP3*, *HACE1*, *HECW2*, *HERC6*, *PJA1*, *RNF114*, *RNF19A*, *RNF34*, *SMURF2*, *TRIM71*, *TRIM9*, *UBE3D*	4.32 × 10^−11^
SUMOylation of MDM2 with SUMO1	1	3	*MDM2*, *PIAS1*, *TRIM27*	2.81 × 10^−6^
Expression of IFNG-stimulated genes	1	4	*MID1*, *TRIM2*, *TRIM22*, *TRIM45*	2.93 × 10^−4^

**Table 6:** Main Reactome *Pathways* overrepresented by ubiquitin E3 ligase adaptor genes depicted in Figure 15.

Group	No. of Reactome Pathways	No. of E3 Adpator Genes in Pathway	Genes	*p* for Group
Neddylation	1	12	*CISH*, *DCAF10*, *DCAF16*, *DCAF7*, *DDB2*, *FBXL13*, *FBXL14*, *FBXO15*, *FBXO31*, *GAN*, *KCTD6*, *KLHL42*	3.46 × 10^−11^
APC/c related pathways	17	3	*ANAPC1*, *ANAPC5*, *ANAPC7*	1.31 × 10^−4^

**Table 7:** Main Reactome *reactions* over-represented by ubiquitin E3 ligase adaptor genes depicted in Figure 14.

Group	No. of Reactome Reactions	No. of Genes in Pathway	Genes	*p* for Group
HCMV nuclear pore docking	1	4	*DYNC1I1*, *DYNC1I2*, *NUP37*, *RAE1*	4.49 × 10^−5^
Neddylation–CUL 4 adaptors	3	4	*DCAF10*, *DCAF16*, *DCAF7*, *DDB2*	3.56 × 10^−5^
Neddylation–CUL1,CUL3,CUL5 adaptors	8	8	*FBXL13*, *FBXL14*, *FBXO15*, *FBXO31*, *GAN*, *KCTD6*, *KLHL42*, *CISH*	1.71 × 10^−9^
APC/c related pathways	37	3	*ANAPC1*, *ANAPC5*, *ANAPC7*	5.28 × 10^−3^

**Table 8:** Major over-expressed pathways associated with differential expression of UPS genes in subtypes of ependymoma.

UPS Component	Pathways	UPS Genes
Ubiquitin activators	Neddylation,	UBA3
	Activation of UBE2Z	UBA6
Ubiquitin conjugases	Antigen processing: ubiquitination and proteasome degradation	Many (Table 2)
	APC/c complex and cell cycle	UBE2C, UBE2S
	DNA stability and repair	UBE2T
	SUMOlyation	UBE2I
	Neddylation	UBE2M, UBE2F
	* Fatylation	UBE2Z
Ubiquitin ligases	Antigen processing: ubiquitination and proteasome degradation	Many (Table 4)
	Cell cycle regulation	APC/c, CDC20
	Induction (or inhibition) of interferon	HDAC4, PIAS1
	SUMOlyation	PIAS1
	Notch signaling	HDAC4, DTX1, DTX4
	Regulation of Hox genes	RNF 20, RNF40
Ubiquitin ligase Adaptors	Neddylation	Many (Table 6)
	APC/c related pathways	ANAPC1, ANAPC5, ANAPC7

* addition of FAT10 (aka Ubiquitin D), a ubiquitin like modifier, to a target protein [133].

## Data Availability

The data referred to in this manuscript are publicly available at the R2 Genomics Analysis and Visualization Platform (http://r2.amc.nl, accessed on 10 January through 21 August 2022) and are available on reasonable request from the first author.
